# Laparoscopic cholecystectomy for duplication of the gallbladder: A case report

**DOI:** 10.1016/j.ijscr.2025.111487

**Published:** 2025-06-07

**Authors:** Yuntian Liu, Xusheng Yang, Lu Liang, Bihui Yao, Xiaoqin Yang

**Affiliations:** a Affiliated Baotou Clinical College of Inner Mongolia Medical University, IC 014040, Inner Mongolia, China; b Hepatobiliary Surgery Department, Baotou Central Hospital, IC 014040, Inner Mongolia, China

**Keywords:** Gallbladder malformation, Duplicated gallbladder, Laparoscopic cholecystectomy

## Abstract

**Introduction:**

Gallbladder duplication is a rare anomaly among clinical gallbladder diseases, with an incidence of approximately 1: 4000 worldwide. Abnormal embryonic development can lead to gallbladder duplication and alterations in the morphology of these structures. Currently, laparoscopic cholecystectomy remains the preferred treatment for gallbladder duplication. However, due to its anatomical variations, it is easy to cause iatrogenic damage during surgery.

**Presentation of case:**

Here, we report the case of a 36-year-old female patient who was referred for recurrent biliary colic and underwent laparoscopic cholecystectomy, as scheduled. A duplicated gallbladder was found intraoperatively. We successfully removed both of the gallbladders. The patient recovered uneventfully and was discharged four days after surgery.

**Discussion:**

Due to its rarity, gallbladder duplication is seldom identified preoperatively due to various confounding factors; consequently, this condition is frequently diagnosed during surgery. Due to its anatomical variations, gallbladder duplication poses a significant risk of iatrogenic injury during surgical procedures, thus representing a significant challenge to the surgeon's experience and expertise.

**Conclusion:**

Laparoscopic ultrasound (LUS) and indocyanine green-fluorescent cholangiography (ICG-FC) can help reduce the risk of vascular injury and enhance surgical safety, especially in patients with gallbladder malformations or when the anatomy of Calot's triangle is unclear due to severe inflammation.

## Highlights

• Gallbladder duplication is a rare anomaly with low clinical incidence and is often difficult to detect preoperatively.
• Surgical treatment of gallstones in patients with duplicated gallbladder malformations is technically complex.
• Integrating LUS, ICG and FC can reduce intraoperative complications and assist surgeons in performing safer operations.

## 1 Introduction

Gallbladder duplication is relatively rare among clinical gallbladder diseases, with an incidence of only 1:4000 worldwide [1]. The primary cause of this anomaly is the abnormal division of the hepatic diverticulum's caudal bud during embryonic development. Currently, the clinical classification mainly follows the Harlaftis system, which includes Type I (single cystic duct), Type II (two cystic ducts independently draining into the common bile duct), and Type III (complex variations). Most patients are asymptomatic but may present with typical biliary colic when complicated by gallstones or inflammation. Although ultrasonography is a common diagnostic modality for gallbladder disease, there is a high possibility of missed diagnosis. Therefore, confirmation often requires the combination of ultrasonography with magnetic resonance cholangiopancreatography (MRCP), endoscopic retrograde cholangiopancreatography (ERCP), and intraoperative exploration. Laparoscopic cholecystectomy remains the preferred treatment method for gallbladder duplication. However, it remains necessary to be vigilant about the risk of iatrogenic bile duct injury resulting from complex anatomical variations.

This case report has been reported in line with the SCARE Criteria [2].

## 2 Presentation of case

**Fig. 1:**
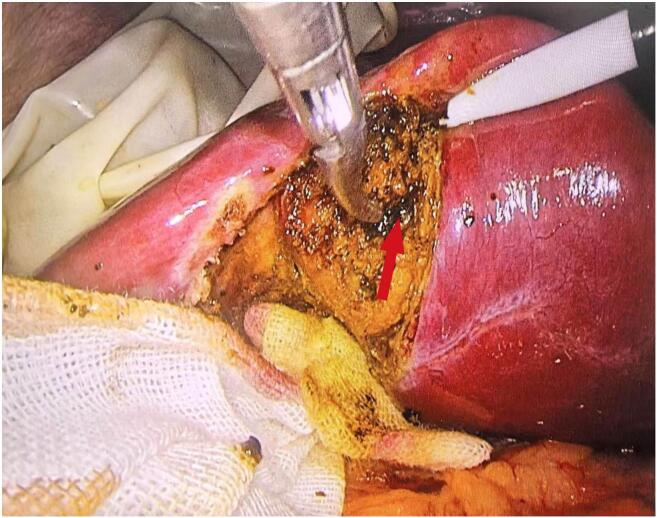
The connection between the primary gallbladder and the secondary gallbladder (red arrow). (For interpretation of the references to colour in this figure legend, the reader is referred to the web version of this article.)

**Fig. 2:**
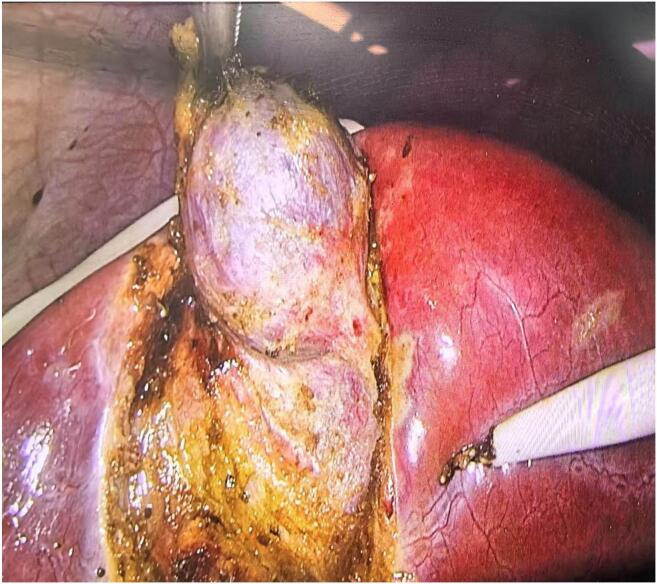
The secondary gallbladder dissected from the gallbladder bed.

**Fig. 3:**
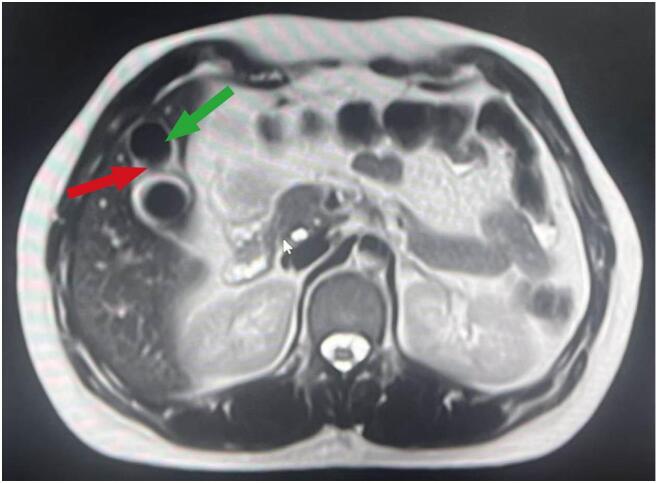
The secondary gallbladder (green arrow) and the connection between the primary gallbladder and the secondary gallbladder (red arrow). (For interpretation of the references to colour in this figure legend, the reader is referred to the web version of this article.)

A 36-year-old female presented to our hospital with episodic upper abdominal discomfort lasting for more than one month, which typically occurred after consuming greasy food. These episodes manifested as a distending pain accompanied by radiating pain to the lower back. The patient experienced intermittent nausea but no vomiting. She reported no fever, chills, cough, sputum production, chest tightness, or shortness of breath. On admission, physical examination revealed the patient was alert and oriented, with no jaundice observed in the skin, mucous membranes, or sclera. The abdomen was soft without tenderness, rebound pain, or muscle guarding. Murphy's sign was positive; percussion tenderness in the hepatic and renal regions was negative. The patient had no significant past medical history. Abdominal ultrasound (US) indicated cholecystitis, multiple gallbladder stones, and a fatty liver. Preoperative routine examinations, including electrocardiogram (ECG), cardiac ultrasound, and chest computed tomography (CT) showed no obvious abnormalities. Preoperative blood biochemical tests revealed abnormal liver function: ALT 132 U/L and AST 117 U/L. Given the possibility of transient common bile duct stones, further evaluation with MRCP was conducted. MRCP findings showed a gallbladder of normal size and shape, but with thickened and irregular walls, as well as nodular short T2 signals within the gallbladder lumen, indicative of multiple gallbladder stones and cholecystitis. The preliminary diagnosis was gallbladder stones with chronic cholecystitis. In accordance with clinical guidelines [3], the patient met the surgical indications and had no apparent contraindications. A three-port laparoscopic cholecystectomy was performed on the third day after admission. During surgery, the gallbladder measured approximately 10 × 8× 6 cm^3^. The gallbladder was gradually dissected from its bed along the serosal layer. Following successful removal, a small amount of bile leakage from the gallbladder bed was noted (Fig. 1). Upon further careful exploration, a cystic structure measuring approximately 4 × 3 × 3 cm^3^ embedded in the liver was observed on the left side of the gallbladder bed. Given its rich surrounding vascularity, a secondary gallbladder was suspected (Fig. 2). This structure was carefully dissected and removed from the gallbladder bed, followed by electrocautery hemostasis of the gallbladder bed. Postoperative examination of the specimen revealed a single cystic duct but two separate gallbladder bodies. The wall of the main gallbladder was slightly thickened and contained five stones, each approximately 2 cm in size. The wall of the accessory gallbladder measured approximately 0.1 cm in thickness and contained a single 3 cm stone. A septum was observed separating the two gallbladders. A careful postoperative review of the MRCP images suggested the presence of a duplicated gallbladder (Fig. 3). Pathological analysis confirmed chronic inflammation with gallstones in both gallbladders. The patient recovered without complications and was discharged four days after surgery.

## 3 Discussion

Gallbladder duplication is a relatively rare clinical gallbladder disorder with an incidence of approximately 1:4000 worldwide. Most surgeons will never encounter such cases throughout their entire careers, and the existing literature relates to rare case reports. Common clinical gallbladder anomalies include: (1) abnormalities in the number, size, or shape of gallbladders, including agenesis, duplication, triplication, giant gallbladder, and gallbladder diverticulum; and (2) positional anomalies, such as left-sided, intrahepatic, suprahepatic, retroverted, folded, and floating gallbladders [4].

During the fourth week of human embryonic development, a hepatic diverticulum emerges from the primitive midgut wall. This diverticulum is the embryological origin of the liver, extrahepatic biliary ducts, gallbladder, and pancreas. Two bud-like structures arise from this hepatic diverticulum: the cranial bud, which further develops into the liver and extrahepatic bile ducts, and the caudal bud, which subdivides into the superior and inferior buds. The inferior bud eventually gives rise to the ventral pancreas, while the superior bud develops into the gallbladder and cystic duct [5]. Abnormal division of this bud-like structure during early embryonic stages can result in duplication of the gallbladder or the formation of additional biliary structures. Furthermore, genetic mutations or hereditary factors can similarly influence gallbladder development by interfering with the differentiation of bud-like structures or altering the normal morphology of the gallbladder. Concurrently, external environmental factors, including maternal infections, malnutrition, or exposure to certain teratogenic agents during pregnancy, may further disrupt the normal embryological development of the gallbladder and biliary system, thus leading to structural anomalies. The exact pathogenesis of this malformation remains unclear; however, further research into the embryological development of the biliary tract may improve our understanding of its anatomical characteristics, thus providing a theoretical basis for clinical diagnosis and treatment.

**Fig. 4:**
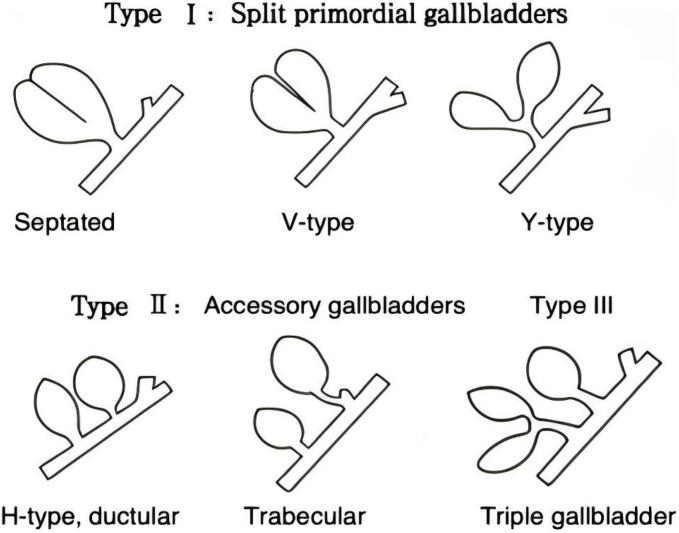
Harlaftis classification for anatomical variations of the gallbladder.

As early as 1926, Dr. Boyden was the first to report gallbladder duplication following cadaveric dissection and radiological investigation. Boyden classified this anomaly into three types: Type I (septate gallbladder), where two gallbladders communicate partially or completely and share one common cystic duct; Type II (Y-shaped duplication), in which two separate gallbladders without internal communication possess individual cystic ducts that merge before entering the common bile duct; and Type III (H-shaped duplication), comprising two entirely separate gallbladders, each with an independent cystic duct entering separately into the common bile duct. Of these, Types Y and H are the most common [6]. Subsequently, Gross et al. [7] and Harlaftis et al. [8] further refined this classification. Currently, the Harlaftis classification is widely used clinically and includes: Type I (comprising septate, V-shaped, and Y-shaped gallbladders, characterized by a single cystic duct entering the common bile duct); Type II (characterized by the main and accessory gallbladders each draining independently via separate cystic ducts into the common bile duct); and Type III (encompassing anatomical variations that do not match Types I or II, such as triple gallbladder anomalies or other rare abnormalities) [9] (Fig. 4).

Most patients with gallbladder duplication typically have no obvious symptoms or may present with only nonspecific gastrointestinal symptoms, such as occasional mild upper abdominal discomfort, bloating, poor appetite, and changes in stool consistency. These symptoms are easily overlooked or misdiagnosed as more common gastrointestinal disorders such as gastritis, gastric ulcers, or functional dyspepsia. When pathology arises in one or both gallbladders, such as cholecystitis or gallstones, patients may exhibit characteristic symptoms, including episodic right upper abdominal pain, which can manifest as colicky, dull, or distending pain, often radiating to the right shoulder or back, these symptoms may be frequently accompanied by nausea and vomiting. In this case, the patient presented due to episodic upper abdominal discomfort that worsened after consuming greasy foods. Nevertheless, given the rarity of gallbladder duplication, clinicians rarely consider this diagnosis initially, even when such symptoms occur.

Although the US is commonly utilized to diagnose gallbladder diseases, the specific diagnosis of gallbladder duplication remains challenging. Firstly, due to the low incidence of gallbladder duplication and limited clinical experience, ultrasonographers are prone to missed diagnoses or misdiagnoses. Secondly, a number of factors can influence the clarity and accuracy of US images, including anatomical variations, inflammation, and gallstones. Furthermore, cases that are suspected of gallbladder duplication by US are often associated with low diagnostic confirmation rates following surgical exploration [10]. Thus, without typical clinical features, US alone cannot reliably diagnose gallbladder duplication. MRCP is currently the imaging modality of choice for suspected gallbladder duplication due to its ability to visualize the biliary anatomy in high resolution [11]. However, even MRCP is not entirely reliable for the diagnosis of gallbladder duplication. This is because of diagnostic accuracy of MRCP can be influenced by a range of factors, including slice thickness, scanning techniques, and the complexity of anatomical variations. ERCP is considered by some researchers as the gold standard for diagnosing gallbladder duplication [12]. However, the invasive nature of ERCP and the associated risk of severe complications create significant limitations for its routine clinical application for diagnostic purposes. Furthermore, the relatively high costs of MRCP and ERCP limit their widespread application as routine diagnostic methods.

Gallbladder duplication must be differentiated from various other gallbladder anomalies, particularly gallbladder diverticula or folded gallbladder. Gallbladder diverticula presents as localized and outward-protruding sac-like structures of the gallbladder wall, typically communicating with the gallbladder lumen. Upon imaging, these sac-like structures appear as localized projections of the gallbladder wall and can be easily confused with Boyden type I gallbladder duplication. A folded gallbladder, resulting from external or internal anatomical factors (such as fibrous tissue or excessive muscular development), manifests as partial or complete folding of a single gallbladder. Although these anomalies may exhibit overlapping imaging features, clear differentiation can be achieved by combining imaging techniques with intraoperative anatomical confirmation. An accurate diagnosis therefore requires the comprehensive evaluation of clinical manifestations, imaging findings, and other relevant diagnostic results, placing significant demands on clinical experience and expertise.

Laparoscopic cholecystectomy remains the first-line treatment for gallbladder duplication [13], with most experts recommending simultaneous removal of both gallbladders during surgery, even if one remains functional. This strategy is performed to prevent postoperative complications such as recurrent biliary colic, recurrent gallstones, biliary pancreatitis, or other serious complications [14]. Many clinical approaches have been investigated in an attempt to address the challenging nature of the biliary anatomy and accurately identify the common bile duct. Intraoperative ultrasound (IOUS) is widely employed in oncologic and transplant surgeries due to its convenience, lack of radiation exposure, and low cost. Zacherl J et al. reported that the sensitivity of IUS for detecting colorectal liver metastases was 95.2 %, the highest among all diagnostic modalities, including CT and MRI. It has even been proposed as the gold standard for evaluating liver tumors [15]. With advancements in medical technology, the integration of IOUS and laparoscopic techniques has led to a technique referred to as laparoscopic ultrasound (LUS), which has been validated for its efficacy in identifying the gallbladder-common bile duct junction [16]. Despite its potential benefits in LC, the routine use of LUS remains limited, with only about 1 % of surgeons adopting the technique. It is primarily employed in cases where the anatomy of Calot's triangle is obscured by inflammation or when gallbladder anomalies are suspected preoperatively [17]. In recent years, a growing body of research has supported the use of indocyanine green (ICG) fluorescent cholangiography (FC) in combination with laparoscopic surgery. This approach enables surgeons to clearly visualize the extrahepatic biliary tract prior to dissecting Calot's triangle [[18], [19], [20]]. A recent review also indicates that intraoperative ICG-FC can assist surgeons in evaluating anatomical structures and selecting safer surgical strategies, although standardized clinical guidelines have yet to be established [21].

## 4 Conclusion

Gallbladder duplication is a rare anatomical anomaly with a very low clinical incidence and is often difficult to detect preoperatively due to a variety of confounding factors. Consequently, this condition is sometimes diagnosed intraoperatively. Because gallbladder duplication has multiple anatomical variants, this condition is associated with specific risks of iatrogenic injury during surgery, thus challenging the skill and expertise of clinicians. However, integrating LUS, ICG and FC can effectively reduce the occurrence of intraoperative complications and assist surgeons in performing safer operations.

## Author contribution

Yuntian Liu: Data curation, Writing- Original draft preparation.

Xusheng Yang: Writing- Reviewing and Editing.

Lu Liang: Conceptualization and Supervision;

Bihui Yao: Visualization, Investigation;

Xiaoqin Yang: Validation Methodology and Software;

All authors read and approved the final manuscript.

## Consent to participate

Written informed consent was obtained from all individual patients included in the study.

## Ethical approval

This is a case report. The Baotou City Central Hospital Research Ethics Committee has confirmed that no ethical approval is required.

## Guarantor

Yuntian Liu.

## Research registration number

Not applicable.

## Funding

This research did not receive any specific grant from funding agencies in the public, commercial, or not-for-profit sectors.

## Conflict of interest statement

The authors declare that they have no known competing financial interests or personal relationships that could have appeared to influence the work reported in this paper.

## Data Availability

The datasets of the present study are available from the corresponding author upon request.
